# A qualitative exploration of allied health providers’ perspectives on cultural humility in palliative and end-of-life care

**DOI:** 10.1186/s12904-023-01214-4

**Published:** 2023-07-12

**Authors:** Hardeep Singh, Arta Taghavi Haghayegh, Riya Shah, Lovisa Cheung, Sachindri Wijekoon, Kevin Reel, Ruheena Sangrar

**Affiliations:** 1 Department of Occupational Science & Occupational Therapy, Temerty Faculty of Medicine, University of Toronto, 500 University Avenue, Toronto, ON M5G 1V7 Canada; 2 Rehabilitation Sciences Institute, Temerty Faculty of Medicine, University of Toronto, Toronto, Canada; 3 KITE-Toronto Rehabilitation Institute, University Health Network, Toronto, Canada; 4 Department of Physical Therapy, Temerty Faculty of Medicine, University of Toronto, Toronto, Canada; 5 Ethics Centre, Sunnybrook Health Sciences Centre, Toronto, Canada

**Keywords:** Cultural humility, Qualitative, Allied health, Palliative care, End-of-life

## Abstract

**Background:**

Cultural factors, including religious or cultural beliefs, shape patients’ death and dying experiences, including palliative and end-of-life (EOL) care preferences. Allied health providers must understand their patients’ cultural preferences to support them in palliative and EOL care effectively. Cultural humility is a practice which requires allied health providers to evaluate their own values, biases, and assumptions and be open to learning from others, which may enhance cross-cultural interactions by allowing providers to understand patients’ perceptions of and preferences for their health, illness, and dying. However, there is limited knowledge of how allied health providers apply cultural humility in palliative and EOL care within a Canadian context. Thus, this study describes Canadian allied health providers’ perspectives of cultural humility practice in palliative and EOL care settings, including how they understand the concept and practice of cultural humility, and navigate relationships with patients who are palliative or at EOL and from diverse cultural backgrounds.

**Methods:**

In this qualitative interpretive description study, remote interviews were conducted with allied health providers who currently or recently practiced in a Canadian palliative or EOL care setting. Interviews were audio-recorded, transcribed, and analyzed using interpretive descriptive analysis techniques.

**Results:**

Eleven allied health providers from the following disciplines participated: speech-language pathology, occupational therapy, physiotherapy, and dietetics. Three themes were identified: (1) Interpreting and understanding of cultural humility in palliative and EOL care (i.e., recognizing positionality, biases and preconceived notions and learning from patients); (2) Values, conflicts, and ethical uncertainties when practicing cultural humility at EOL between provider and patient and family, and within the team and constraints/biases within the system preventing culturally humble practices; (3) The ‘how to’ of cultural humility in palliative and EOL care (i.e., ethical decision-making in palliative and EOL care, complexities within the care team, and conflicts and challenges due to contextual/system-level factors).

**Conclusions:**

Allied health providers used various strategies to manage relationships with patients and practice cultural humility, including intra- and inter-personal strategies, and contextual/health systems enablers. Conflicts and challenges they encountered related to cultural humility practices may be addressed through relational or health system strategies, including professional development and decision-making support.

**Supplementary Information:**

The online version contains supplementary material available at 10.1186/s12904-023-01214-4.

## Background

Palliative and end-of-life (EOL) care periods can be highly stressful for patients and their families as they are associated with difficult issues related to death and dying [1–3]. Individuals have unique experiences of palliation based on the beliefs and practices of the communities they belong to [4–10]. However, irrespective of individual approaches to palliation, patients and families share some common emotions. For example, patients receiving palliative care have reported feelings of loss, worry, fear, and other non-physical forms of suffering [11]. These emotions may be reciprocally related to their experience of functional loss [12], lower quality of life [13, 14], loss of meaning in life [15], and decreased appetite and sleep [16].

In palliative and EOL care, allied health providers (e.g., occupational therapists, physiotherapists, speech-language pathologists, dietitians, occupational therapy assistants, and physiotherapy assistants) provide specialized support and interventions, such as rehabilitation, to address physical, emotional, and functional needs to enhance the overall quality of life of patients and families [17–21]. Allied health services aim to help patients maximize their well-being and independence for as long as possible, maximize their functional status, and improve their quality of life by minimizing the physical, psychological, and spiritual symptoms associated with life-threatening diseases [17–20, 22]. Moreover, allied health services are integral in helping patients with advanced diseases restore or maintain their engagement in meaningful activities [14, 20, 23].

Within palliative and EOL care, allied health providers who differ in language and culture from their patients may encounter challenges or tensions, such as language barriers, diverse expectations between patients from similar backgrounds, and the risk of stereotyping patients needs based on their cultural identities [24, 25]. Individuals from underrepresented and marginalized communities can experience a lack of culturally appropriate communication [26–28], lower quality of EOL care [29–31] and poor patient-provider relationships [32, 33]. Allied health providers of cultures different from their patients may not understand the patient’s cultural beliefs and preferences regarding their palliative experiences [24, 33–36]. Culture is a multidimensional and dynamic concept [37], defined as “learned, shared and transmitted values, beliefs, norms and lifeways of a particular group that guides their thinking, decision, actions in partnered ways” [38, 39]. Culture can encompass multiple factors, such as race, ethnicity, gender-identity (i.e., a person’s internal and individual experience of gender” [40, 41]), age, sexual orientation, religion, and socioeconomic factors [42, 43]. Cultural factors, including religion or religious beliefs, can shape the meanings, beliefs, and decisions patients associate with death and dying and impact palliative and EOL care needs (e.g., communication preferences) [4–10]. A meta-synthesis examining 16 EOL care studies found that patients and family members generally described effective communication preferences in palliative care as being carried out through shared decision-making, respectful and compassionate care, and enabling choices [44]. Health providers must also acknowledge and attend to the communication preferences of individuals from cultures and religions that may differ from their own, such as preferences for ‘bad news’ communication, conversations related to health decision-making processes (e.g., collective versus individual), and attitudes related to advance directives and EOL care [10]. For instance, some patients and families may find it culturally inappropriate for a health provider to discuss a life-threatening diagnosis directly with the patient without the family or an authoritative member of the family present [33].

A poor understanding of a patient’s and/or family member’s cultural beliefs and preferences can negatively impact the relationship between the patient and clinician [33] and result in EOL care not being tailored to the patient’s needs or causing harm [45]. Given the importance of culture in grief, death, and dying, allied health providers are encouraged to understand and attend to the cultural needs of patients and families in clinical encounters [4, 46–48]. Allied health providers who recognize a patient’s cultural needs, preferences, values, and beliefs can better support individuals experiencing the dying process [26, 33]. However, knowledge gaps in navigating relationships and practice with culturally diverse patients in palliative and EOL care settings may prevent allied health providers from consistently integrating patient and family members’ cultural needs into EOL care [45]. A qualitative study by Milberg & colleagues in 2016 explored health providers’ (n = 60) understanding of cross-cultural interactions during EOL care [49]. While health providers in this study had limited cross-cultural interactions in EOL care, they experienced uncertainty, stress, and concerns about their ability to provide equitable EOL care during cross-cultural interactions [49].

Cultural humility is described as an ongoing ‘mindset’ or practice wherein healthcare providers self-evaluate their assumptions, biases, and values, which allows them to foster an understanding of other individuals’ preferences [50, 51]. As such, cultural humility differs from prescriptive approaches, such as culturally competent care guidelines that some scholars have criticized in palliative and EOL care due to concerns of negative impacts on care (e.g., leading to stereotyping and preventing holistic care) [49]. Cultural humility has been promoted by allied health providers’ professional organizations [52–54] and organizations in palliative and EOL care [55, 56] as an essential approach. This approach may help optimize palliative care by ensuring healthcare providers critically examine their awareness and understanding of a patient’s cultural beliefs and practices and encourage collaboration to determine their needs and preferences for EOL planning, decisions and goals [50, 57–60]. Continuous consultation with patients and families, oneself, and other healthcare providers may help providers assess the cultural dimensions of the patient’s experience, manage power dynamics, and ensure patients are equal partners in the therapeutic alliance [4, 46, 47, 51, 61].

While cultural humility is an important aspect of allied health practice, there are knowledge gaps regarding how rehabilitation practitioners demonstrate it in palliative and EOL care. To our knowledge, allied health providers’ perspectives of cultural humility and how they apply it in palliative and EOL care within a Canadian context have not been examined. Examining the Canadian context is particularly necessary as Canada is a culturally diverse country with over 450 ethnic and cultural communities reported in the 2021 census data [62], and ethnocultural diversity is expected to grow in Canada due to continuing immigration over the next decades [63]. This foundational knowledge is necessary to reduce the challenges, such as uncertainty and stress, allied health providers experience during cross-cultural care provision, to inform a higher quality of palliative and EOL patient care, and to contribute to reducing palliative care disparities [64, 65].

## Methods

### Aim

This qualitative study aimed to describe Canadian allied health providers’ perspectives of cultural humility practice in palliative and EOL care settings. The following research questions guided this study:

**1**) How do allied health providers working in palliative and EOL care understand the concept of and practice cultural humility?

**2)** How do allied health providers navigate relationships with patients who are palliative or at EOL and from differing cultural backgrounds?

### Design

This qualitative study employed an interpretive description (ID) design [66] that is well-suited for qualitative research that aims to study a clinical phenomenon and generate actionable insights that can guide clinical practice [66]. As such, this methodology was appropriate to answer our research questions as it aims to address clinical problems by exploring associations and relationships across data using disciplinary logic [66]. Ethics approval was obtained from the University of Toronto’s Research Ethics Board (#42,992) and all methods were performed in accordance with the relevant guidelines and regulations. The Standards for Reporting Qualitative Research checklist was used to ensure comprehensive reporting [67].

### Characteristics of participants and setting of study

We aimed to recruit a sample size of 15–20 participants. This target sample was informed by the study objectives, resources [66], prior ID studies in palliative care [68–70], and the concept of data sufficiency (i.e., sufficient data to answer research objectives) [71]. Participants were recruited from across Canada using purposeful sampling strategies, including emailing recruitment flyers to palliative care providers, organizations, and clinical groups [72]. Individuals were eligible to participate if they were allied health providers (e.g., occupational therapists, recreational therapists, physiotherapists, speech language pathologists, dietitians, or occupational therapy/ physiotherapy assistants) who currently or recently (within the past five years) practiced in a Canadian palliative or EOL care setting.

### Data collection

Individual interviews were conducted remotely on Zoom (n = 9) or over the phone (n = 2) based on the participant’s preference by authors ATH and HS. The interviews were audio recorded and transcribed verbatim. Demographic characteristics, such as age, profession, years as a clinician, and years working in palliative care, were collected to describe the study sample. Using a semi-structured interview guide, each interviewer encouraged participants to share examples from their clinical practice that illustrated their viewpoints. The guide was iteratively updated based on preliminary data analysis from concurrent data collection and analysis (see supplementary material for the interview guide). Interviews ranged from 40 to 66 min (an average of 56 min).

### Data analysis

Data from the transcripts and memos were analyzed inductively using strategies outlined by Thorne (2016) [66]. First, two researchers (HS and ATH) independently listened to the audio recordings, read the transcripts, and reviewed their memos to immerse themselves in the data. Potentially meaningful quotes and perspectives were flagged, and contrasting viewpoints were highlighted. Transcripts were then imported to NVivo version 12. HS and ATH independently organized data in broad codes related to the research questions to examine data from various perspectives while refining and reconstructing patterns within these codes to meaningfully make sense of the patterns. After independently coding six transcripts, HS and ATH met to discuss, compare, and combine their codes, and to create a refined codebook. ATH used the refined codebook to code all remaining transcripts. After all transcripts were coded, HS and ATH combined similar codes and formed preliminary themes. Finally, to enhance rigour, LC, who is experienced in palliative and EOL care, reviewed three transcripts and provided further interpretations, which were compared and integrated into the final themes with input from the entire research team.

### Trustworthiness

We employed strategies outlined in ID to enhance research trustworthiness [66]. First, epistemological integrity, logic, and methodological coherence between the research questions, methods, and analysis, and the research team’s interpretations were enhanced by reflecting on how their personal and clinical experiences and disciplines may impact their interpretations. For example, investigators with personal experience of palliative care considered how their own interactions with allied health providers aligned or were contrary to the perspectives raised by participants. In addition, ATH and HS created memos during the interviews to capture their thoughts, reflections, and feelings about the discussions. Second, representative credibility, or the alignment between the knowledge claims and the data, was enhanced by interviewing multiple participants and including multiple research team members in the analysis. Third, the researcher’s interpretations of the themes were supported with verbatim participant quotes in the report. Fourth, interpretive authority, the confidence that the researchers’ interpretations are trustworthy, was enhanced by identifying verbatim participant quotes to support researchers’ interpretations and involving multiple research team members, including those with personal and clinical experiences in palliative care, in the analysis. Finally, the Standards for Reporting Qualitative Research checklist was used to improve reporting transparency [67].

### Positionality

Interpretivist constructivist perspectives underpinned this study as ID views researchers as an interpretive instrument for building research insights and the existence of multiple realities [66]. The research team included individuals who were physiotherapist (LC) and occupational therapists (HS; RuS; SW; KR), a student occupational therapist (RiS) and a rehabilitation research trainee (ATH). The research team self-identified with the following ethnicities: South Asian (HS; ATH; RSi; RuS; SW), East Asian (LC), and White (KR). The research team stated these gender identities: women (LC; HS; RSi; RuS; SW) and men (ATH, KR). In addition, many of the research team members had prior research, personal, or clinical experiences with palliative and EOL care. Many of the research team members have examined concepts of cultural humility in their practice and teaching to align with personal values and professional obligations.

## Results

Eleven participants participated in this study (see Table 1 for participant characteristics). Participants belonged to the following professions: speech-language pathology (n = 1), occupational therapy (n = 6), physiotherapy (n = 3), and dietetics (n = 1). Participants had an average of 6.6 years of experience working in palliative/EOL care within an overall average of 12 years of practice (1–15 years) experience as allied health professionals. Participants were aged 29 to 49 years (average 38 ± 6.4 years). All participants identified as women except for one who identified as non-binary. Participants self-identified the following ethnicities: East Asian (n = 2), White (n = 7), and South Asian (n = 2). Participants stated the following when asked, “which religion do you identify with (if any)”: secular (n = 1), no religion (n = 5), agnostic (n = 2), Muslim (n = 2), Christian/other (n = 1). Participants described working in the following settings: hospital inpatient units (e.g., palliative care, oncology, intensive care unit) (n = 8), hospital outpatient (e.g., amyotrophic lateral sclerosis clinic) (n = 1), and community in-home care (n = 2).

**Table 1:** Participant demographics and work characteristics as identified by participants

Participant ID	Profession	Years working in palliative care
RT-001	Speech-language pathologist	11
RT-002	Occupational therapist	5
RT-003	Occupational therapist	4
RT-004	Occupational therapist	1
RT-006	Physiotherapist	6
RT-007	Dietitian	2
RT-008	Physiotherapist	15
RT-009	Occupational therapist	9
RT-010	Physiotherapist	1
RT-011	Occupational therapist	15
RT-012	Occupational therapist	4

The following three themes were identified: (i) Interpreting and understanding of cultural humility in palliative and EOL care; (ii) Values, conflicts, and ethical uncertainties when practicing cultural humility at EOL; (iii) The ‘how to’ of cultural humility in palliative and EOL care (see Table 2).

**Table 2:** Themes, subthemes and supporting quotes

Themes	Subthemes	Supporting quotes
**Theme 1: Interpretating and understanding of cultural humility in palliative and EOL care**	Subtheme 1a: Recognizing my positionality, biases, and preconceived notions	“People are going to have had different experiences, different upbringings… the things that you value and think are important and proper are not necessarily going to be the same as them…the humility piece is just acknowledging that there’s more than one way to do things… it can’t be about what you would want or what you would do in a particular situation” (RT-008, physiotherapist). “I am someone who identifies as like moderately religious, I’m kind of like ‘Whoa, whoa’ especially if it’s someone within my same kind of religion; someone who is a version of Christianity…I find it sometimes harder not to fight against it and be like ‘No, that’s not how this works’” (RT-003, occupational therapist).
	Subtheme 1b: Check myself: Learning from patients	“When families don’t want their loved ones knowing about their cancer diagnosis and or prognosis and thinking ‘I would want to have that information’ you worry about them, like, are they hiding stuff? Are they wanting mom to keep fighting and not giving them the full info? But then I learned a little while ago that in some cultures, they’re shielding this information because they don’t want to burden the person and they want them to just be not carrying the weight of knowing prognoses- Like, oh, that’s interesting. I never would have thought of that, so I need to like check myself if a patient family that’s maybe being a bit more secretive in their approach with what info they want the family to learn, it’s not necessarily for sinister reasons. They’re not trying to hide, they’re trying to not burden, so always opportunities to learn different perspectives” (RT-006, physiotherapist).
**Theme 2: Values, conflicts, and ethical uncertainties when practicing cultural humility at EOL**	Subtheme 2a: Values and conflicts between provider and patients and family, and within the team	“Where it can be tricky is specifically when a family will not want you to talk about the fact that a person is palliative; a patient. Because it’s important, in their culture, not to talk about death or dying. It makes it hard to navigate, I guess, open discussion. ‘Cause I often, if I can, to ask a person how much they know about their prognosis and what kinds of choices they’re going to want to be making given how much- Like, how they see their health progressing. And there’s a few families where it’s been very clear, like, don’t mention that you’re from palliative home care. Like, please don’t talk to the client about their diagnosis or their prognosis. And so, it’s uncomfortable” (RT-012, occupational therapist). “Eating is just so important and so central to the culture of the family. There’s, in advanced cancer, I mean, everybody loses their appetite and there’s a phenomenon called anorexia and cachexia…the family, you know, is thinking like ‘Oh my gosh, you’re starving. You’re losing so much weight. You’re not eating. You have no appetite. If you’re ever going to get better, like, you need to eat.’ And so, my role in something like that, like the way that I will approach it is to just affirm…this is not actually a function of their choices, but this is like a part of their disease and something…this is going to be another stage in accepting the fact that their family member is dying” (RT-011, occupational therapist).
	Subtheme 2b: Constraints/biases within the system which prevent culturally humble practices	“I also do have an agenda where I need to also synthesize information quickly because as a clinician – especially in acute care – I don’t really have the time. I don’t have time to make, you know, dedicated appointments. I have a huge- Like, I have to triage appropriately on an ongoing basis because I also have referrals, I also have to balance like my own clinical duties” (RT-001, speech-language pathologist).
**Theme 3: The ‘how to’ of cultural humility in palliative and EOL care**	Subtheme 3a Reflective strategies	“You can’t rid yourself of your biases. I think if you, you know, if you tell yourself that you can, there’s something that you’re going to miss. Like, there’s something that you’re hiding…but it’s about being as aware of them as I can be so I can make sure that those aren’t the things that are in the driver’s seat when I’m making decisions” (RT-011, occupational therapist).
	Subtheme 3b: Relational strategies	“When you work with different cultures when you enter the room – because you know the basis before entering the room – not assuming things which is associated to a specific culture and not approaching them with your biases or any biases you have heard or you have seen; being more neutral. That’s what I understand about cultural humility” (RT-010, physiotherapist). “Let’s say it’s a Chinese family, and so I know that, like, many times there is that preference not to have someone die in a home, I go in but I adjust my language vague enough. So, for example, talking about- I still reference like, you know, ‘At home this is what it would look like: You would need a hospital bed.‘ At home, this is-‘ I’m not saying for when you go home, I’m using a language that’s general enough to, you know, let the family know that I’m not making any assumptions about whether they are going home or not, but that if they want to, that option is still there” (RT-002, occupational therapist). “Be culturally sensitive to different kinds of religions and how it related to speech. One example I will just kind of discuss is kind of like when we’re doing assessment for like Ramadan, we should be more culturally sensitive on like ‘Okay, can I actually assess you? Can I give you something to eat and drink?’ And also, food that’s halal, like, that’s something at least, again, a lot of my- like I said my interventions with food; making sure, okay, I can’t give them- I have to make sure this is, like, kosher or halal before I actually feed them as part of my assessment. So not only that, like I have to even look at my food just to make sure that I’m providing something that’s respectful. Also asking first, also not assuming that, you know, but asking ‘Are you okay? Can I try this? Is there anything that you can’t eat?’ Kind of like treating it like an allergy I guess, not that it is an allergy, but asking for that information openly” (RT-001, speech language pathologist).
	Subtheme 3c: Contextual/health system enablers	“Make sure that I’m doing everything that I can, and also just grounding my decisions in, like, our, you know, policies” (RT-011, occupational therapist).

### Theme 1: interpreting and understanding of cultural humility in palliative and EOL care

This theme describes common elements of cultural humility identified from allied health providers’ descriptions of this concept. All allied health providers indicated that cultural humility was an intrinsic process of recognizing their positionality, biases, and preconceived notions (subtheme 1a). This was done before, during and after engaging in an extrinsic process of learning from their patients (subtheme 1b).

### Subtheme 1a: recognizing my positionality, biases, and preconceived notions

Regarding their positionality within clinical interactions, allied health providers reflected on their cultural identity and how it shaped their worldview, including the relationships they developed with patients. For example, an occupational therapist shared how reflecting on her experience of coming from a small population context and subsequently moving to a larger urban centre presenting more cultural diversity:

Our clinic sees 750 patients across Ontario and I’m from a very very small town in the middle of nowhere initially, so moving to [de-identified city] has been a big growth curve in my professional practice as well as just as far as having that kind of cultural awareness and humility and learning how to work in those environments. So, I think it’s something that I really tried to consider over the last 5 years and tried to build awareness (RT-003, occupational therapist).

Allied health providers also reflected on how their personal experiences of discrimination based on belonging to minoritized or marginalized cultures shaped their approach to interacting with patients and communities where they practiced:

I used to work in [location] as I mentioned up north where it’s definitely more of a rural area versus urban. So people say, ‘You work at the [name of nationality] store, don’t you!’ like, [nationality] restaurant, and I’m like ‘No, I’m a clinician that’s doing my placements here. I don’t work at the [nationality] supermarket, I’m sorry.’ But then it makes you think ‘Okay, this person has not experienced potentially immigration in their community.’…there’s specific experiences that make me think ‘Okay, they haven’t experienced or have been exposed to different cultures the way I have, and that’s okay’ (RT-001, speech-language pathologist).

Another element of cultural humility that participants discussed was recognizing one’s privileges and powers. To practice cultural humility, a physiotherapist expressed the need to recognize how multiple factors, such as one’s occupation, ethnicity, socioeconomic status, and gender, shape one’s interactions with patients who belong to marginalized communities:

You need to take a moment to recognize what privileges may have been extended to you because of your own background, whether- from whatever category you want to look at, whether it’s ethnicity or socioeconomic status or gender or whatever...how is that impacting you as you approach your interactions with people and can you maybe learn from your interactions with those patients and families that will help you to be a more kind and considerate therapist or maybe conscientious therapist in your next interaction with someone who comes from a different background than you (RT-006, physiotherapist).

In terms of “preconceived notions”, allied health providers indicated that cultural humility meant acknowledging their preferences, values, and norms so they could refrain from imposing those on their patients:

I think it [cultural humility] is really about checking your preconceived notions at the door and checking any- Like, your personal preferences…What you would do in their situation and removing that from it and just reminding yourself that, you know, people are going to have had different experiences, different upbringings…the things that you value and think are important and proper are not necessarily going to be the same as them (RT-008, physiotherapist).

Overall, allied health providers indicated that by recognizing such preconceived notions, discriminations, assumptions, and biases, which are an inherent part of a person, they could open themselves to learning from others (e.g., their patients) and take steps to manage these through conscious strategies (theme 3).

### Subtheme 1b: check myself: learning from patients

Allied health providers indicated that cultural humility contradicted the notion of placing themselves in the patient’s shoes and making assumptions about what a patient would want or do. Instead, they believed culturally humble patient-centred care entailed learning from patients because patients have “different experiences, different upbringings,” (RT-008) and different ideas of what is meaningful or important to them. Allied health providers framed cultural humility as a collaborative relationship focused on aspects of care:

The humility piece is just acknowledging that there’s more than one way to do things and I think if you’re going to try and say you’re actually being patient-centered, then it can’t be about what you would want or what you would do in a particular situation (RT-008, physiotherapist).

Allied health providers acknowledged the existence of their preconceived notions about “what is good or true or right or normal” (RT-003, occupational therapist). However, to them, adopting cultural humility meant that they had to recognize that their way of “knowing” or “being” was one of many and “not better than anyone else’s” (RT-003). Instead of imposing their preconceived notions on others, cultural humility necessitated them to learn from patients about their beliefs and values of what was “true, right or normal” (RT-003) concerning one’s rehabilitation care. As described below, cultural humility entailed learning from patients about themselves with an open mind and curiosity:

To me the words cultural humility, they bring up a feeling around just knowing or like the recognition that there’s just so much that we don’t know and there’s so much to learn about people. It brings up this kind of like- like sometimes when I feel curious, I feel like insatiable, like, I just want to know and it’s almost like aggressive, you know? But like when I think about cultural humility, it’s like gentler. It’s like a curiosity…It makes me think about the honour of my job, like, just like how lucky we are to be able to, you know, be welcomed into, like, all kinds of peoples’ homes and be able to support them at this, like, you know, most sensitive time in life” (RT-011, occupational therapist).

While some allied health providers defined cultural humility as more than one way of knowing, others described it as a form of health providers acknowledging that they do not know everything. For example, two occupational therapists explained that adopting an open mindset required dismantling power imbalances between health providers and patients that have dominated many professions. They also reflected on how damaging it could be to make assumptions about patients based on their cultural backgrounds.

When I read cultural humility I immediately thought of… being a good communicator of openness to understanding someone else’s perspective…we need more of that in medicine, like, there’s so much, like, top-down ‘doctor-knows-all’, ‘doctor-knows-best’, ‘we’ll do it my way or the highway’ - especially in light of Canada’s history with Indigenous people (RT-009, occupational therapist).

There’s always the power differential between kind of like a healthcare professional and then a patient. Essentially, there’s this expectation somehow, that we bring knowledge or like the right way of doing things. And I could see where it could be especially potentially damaging…because we, of course, operate out of our own culture, and so we’d have certain expectations or assumptions that, you know, you’re going to want- ‘I would want it to be this way, so, probably you will want it to be that way’” (RT-012, occupational therapist).

Only after allied health providers invested time to learn from patients about their rehabilitation needs could they fulfil their role of supporting patients at EOL in how they wanted to be supported and cared for.

### Theme 2: values, conflicts, and ethical uncertainties when practicing cultural humility at EOL

When practicing cultural humility with patients from cultures other than their own, allied health providers discussed the conflicts and challenges due to: (i) value conflicts both between provider and patient and family, and providers within the team (subtheme 2a), and (ii) ethical uncertainties: constraints/biases within the system which prevent culturally humble practice (subtheme 2b).

### Subtheme 2a: values and conflicts between provider and patient and family, and within the team

Collectively, allied health providers reported several internal conflicts and challenges related to ethical decision making when providing care for patients from cultures other than their own. These challenges and conflicts were created when there were competing priorities and opinions between the patient’s culturally informed preferences and the provider’s or the provider’s professional or ethical standards. For instance, several participants grappled with conflicts when family members from certain cultures requested that the providers refrain from disclosing their terminal diagnosis or prognosis to a patient since their professional and ethical obligations required open and transparent communication for informed consent. An occupational therapist described feeling uncomfortable when exposed to a family’s preferences for not disclosing a patient’s diagnosis and prognosis, given that transparency was a requirement for informed consent:

Where it can be tricky is specifically when a family will not want you to talk about the fact that a person is palliative - a patient - because it’s important, in their culture, not to talk about death or dying… Like, please don’t talk to the client about their diagnosis or their prognosis. And so, it’s an uncomfortable- It feels uncomfortable to have that kind of space sitting there in my practice (RT-012, occupational therapist).

Another internal conflict allied health providers navigated was related to patient preferences to work with health providers of a specific gender. Understanding this preference was challenging for some providers who indicated they firmly believed in gender equality, and these preferences misaligned with that. In addition, allied health providers shared clinical examples of managing challenging situations in patient interactions that may impact cultural humility. For example, a speech-language pathologist expressed discomfort related to potential professional boundary crossing when patients or their family members asked her to share what she would do in their situations regarding a clinical decision. The provider’s concern was that she did not want to influence their decisions; however, some patients become offended if providers did not share their opinion.

Families will actually ask you, like, ‘What would you do if this was your grandma?’ and that I find is the hardest as a clinician…they wanted to know what [the] clinician would have done.… Sometimes- and I will be honest and I’ll say this actually happened to my grandma; we ended up doing this, but this is what she would have wanted. So, I think sometimes families will want your opinion and rather- and me assuming what they should do, they’ll actually ask, like, ‘What would you do?’ And I think that’s actually the most challenging piece that I’ve had to navigate (RT-001, speech-language pathologist).

A prominent challenge that many allied health providers had to manage was misalignments between the EOL preferences/values of patients and that of their family members. For example, some family members had challenges accepting changes in the patient’s health and function as the patient could no longer fulfil their cultural and gender-based roles and expectations.

The wife…her belief was- She has always seen her husband walking, doing all the, you know, heavy work. And she wanted him to keep doing that because she believed that if he doesn’t do that, she’s going to lose him…But as a health professional, you understand that there are limitations with the disease. And the husband had extreme fatigue, but you know, he wanted to respect his wife’s wishes because he felt guilty about it; that he’s not able to do it (RT010, physiotherapist).

Providers described these conflicts and challenges were common, and at times difficult to manage.

### Subtheme 2b: Constraints/biases within the system which prevent culturally humble practices

Contextual factors also presented a challenge that limited providers’ ability to deliver culturally humble care. Allied health providers explained that delivering culturally humble care required resources, including adequate time and sometimes language translation. However, not all participants felt they had adequate time or resources. Despite the availability of interpreters in health care organizations, many participants found it challenging to communicate due to the possibility of misinterpretation that may affect rapport. Moreover, some allied health providers felt they did not have sufficient time to spend with patients, given their large caseloads and limited resources (e.g., staff). Finally, culturally relevant resources for patients were a gap noted by participants that prevented them from effectively engaging in cultural humility in patient interactions:

A couple was on our unit – 2 men – and, I mean, they were so in love, it was so sweet, but one of them was dying and as such we were supporting the spouse in his grief. And all of our resources are about, like, heterosexual marriages. There was nothing with any sort of language around, like, husband and husband type… homosexual spousal partnership in the grief literature that we had on our unit (RT-009, occupational therapist).

### Theme 3: the ‘how to’ of cultural humility in palliative and EOL care

All allied health providers described their strategies to enhance cultural humility in cross-cultural patient interactions. These strategies were categorized into three broad categories, including reflective (subtheme 3a), relational (subtheme 3b), and contextual/health system enablers (subtheme 3c).

### Subtheme 3a reflective strategies

Participants recounted how reflective and intra-personal strategies supported their ability to incorporate cultural humility in their practice. Intra-personal strategies comprised self-reflection and understanding oneself and one’s emotions. Participants used ‘self-reflection’ to understand their positionality, biases, and preconceived notions, which many believed were inevitable. These strategies were practiced in solitude by allied health providers or internally during and after patient interactions. Internal dialogue of thoughts and processes related to practice was used to direct providers’ course of action in their treatment. For example, an occupational therapist described engaging in self-reflection and awareness by checking in with themselves. They asked themselves, “it’s also really important to be like ‘How am I coming across? Am I listening? Or am I just talking?’” (RT-003), to reflect on the interactions that occurred with the patient. Self-reflection of biases was a critical aspect of understanding one’s self and one’s emotions. It was achieved by “just getting to know our biases, self-reflection…Like, just kind of checking ourselves constantly” (RT-011, occupational therapist). Similarly, RT-001, a speech-language pathologist, shared her strategy: “Try to be reflective to say why we have [assumptions] and think about our own discomfort, like why are we having these feelings and kind of reflect on that as a clinician.”

Participants adjusted their mindset to communicate neutrally and objectively, avoiding assumptions and stereotypes deliberately about a patient’s palliative/EOL care preferences. Many allied health providers did so by listening to patients, while considering their scope of practice, and professional and legal requirements.

Remain as objective as possible and just to follow our professional and legal processes…There’s so many different combinations that people can have from their experience culturally, so I’m not going to assume, I’m going to listen (RT-001, speech-language pathologist).

Participants recognized the need to take a moment to be mindful and reflect on their experiences with patients, mitigate the disturbances of biases and assumptions, to effectively utilize a cultural humility perspective in their practice. Overall, it was believed that these reflective strategies, along with relational and contextual strategies, could help them manage their positionality, biases, and preconceived notions to avoid imposing these on their patients.

### Subtheme 3b: relational strategies

Allied health providers alluded to relational and inter-personal strategies to provide care with cultural humility. Inter-personal strategies were applied through acquiring knowledge and learning from others, such as the patient, the patient’s family members or healthcare colleagues.

Allied health providers explained that they sought to understand a patient’s culture concerning their rehabilitation goals, values and preferences to inform palliative and EOL care rather than generally asking about their culture explicitly. Some allied health providers engaged in discussions with patients about their belongings in their environment (e.g., photographs or other meaningful items) to build rapport and obtain a better understanding of the patient as a person.

Allied health providers noted that having a “general idea” of various cultures’ preferences, norms, customs, and values related to palliative and EOL care and then looking for variation helped inform interactions with patients from different cultures. For example, a dietitian advised, “Familiarize yourself with the wide range of how people observe, you know, different beliefs…Not being too rigid-minded, but just to have a general idea” (RT-007, dietitian). Allied health providers shared examples of cultural preferences related to: preferences among some cultural communities for the location of dying (e.g., some cultures prefer dying at home whereas others consider it a “taboo or a stigma” (RT-002); foods or drinks; communication about prognosis and diagnosis; resuscitation orders; EOL practices (e.g., prayers, visit from a spiritual guide); and decision-making processes (e.g., individual or group-based with family). Providers used this general information to inform their interactions with all patients. For instance, after noting that some cultures engaged in quiet prayer time, a physiotherapist ensured that they always asked each patient when a good time would be for the clinical visit. An occupational therapist indicated they would offer families a private room to grieve as some cultural communities grieved more openly and observably than others (e.g., grief that is more audible, intensely emotional, and expressed in the company of others). However, despite noting these trends, they were careful not to assume all patients of a particular culture held those same specific beliefs. Through a conscious effort, they refrained from informing their clinical thinking and actions with stereotypical assumptions or judgements based on their biases and past experiences:

I try not to, sort of, inform my interventions in the way I interact with my patients based on, like, based on stereotypes but just, sort of, I don’t know a better way to put it, but just sort of reading the room. Like, just, sort of, walking in and meeting people and seeing how they interact (RT-011, occupational therapist).

Many allied health providers commented on how they were deliberate in their interactions with patients to ensure that they were not imposing their perspectives. One common strategy used was asking patients open-ended rather than leading or assumptive questions. As an example, a speech-language pathologist discussed the open conversations they had with a patient to learn more about their food preferences at the end of their life. Similarly, an occupational therapist explained,

Instead of saying, you know, like, ‘Oh does your husband live with you?’, right? Because that becomes a very close-ended question, and even if the answer is yes because I have assumed the person’s sexuality and everything correctly…I’m trying to be more open-ended with my questions (RT-003, occupational therapist)

Allied health providers agreed that incorporating cultural humility in their practice required a team-based approach. Consulting with their colleagues was an essential strategy used by many to gain information on their patients and unfamiliar cultural practices and navigate conflict, challenges, and uncertainties. For example, a physiotherapist discussed the value of their team on their learning: “Working with [name of de-identified] community was a different experience because understanding their beliefs, respecting their beliefs, was different experience for me. In fact, I guess again I would bring my team in here, because there was a lot of help – the chaplain we have; she really educated me on their culture” (RT-010, physiotherapist). Furthermore, participants discussed maximizing resources amongst the professional team. Collaboration amongst the team improved their understanding of cultural needs and facilitated rapport with the patients’ families to support them better. For example, one occupational therapist described the team’s attempt to give families a safe space to accommodate cultural needs, where she mentioned:

Some cultures, they grieve really loudly, and I don’t want to say demonstratively, but it’s more audible - than others do. So if we recognize that this is happening or about to happen, we have like a quiet room so any member of the team can- will go into the patients’ room to say ‘Oh, we have a private space’” (RT-002, occupational therapist).

In sum, inter-personal strategies entailed collaborating with others, including the patient, family members or the team, which was necessary to provide culturally humble care.

### Subtheme 3c: contextual/health system enablers

Other strategies providers used to practice cultural humility stemmed from the contextual environment and the health systems resources available to the allied health providers. One occupational therapist shared that they used search engines, online cultural videos, and evidence-based literature to gain insight into specific cultures, practices, and rituals. In addition, several allied health providers discussed the usefulness and necessity of interpreters on the unit to augment communication. For example, one allied health provider described how using an interpreter when communicating with patients was an important strategy to encourage optimal communication regarding their care. Alternatively, if resources were limited, providers used assistance from the patient’s family members to translate information for the patient. Another occupational therapist reported the use of telephone interpretation services on the unit, as they are accessible and convenient ways of communicating with the patient and their family during treatment and assessments:

It makes things so much easier because you may think that you can just mime your way or use basic English to communicate with whoever- with the patient...but you never get nearly as much information as you do when you’re able to communicate with that patient in their preferred language (RT-002, occupational therapist).

The provider reflected on ensuring the patient and family were aware of the use of interpreters and that it is available for use upon their consent. The availability of on-unit training and education regarding cultural humility in palliative and EOL care is an asset to improving allied health provider knowledge of cultural humility. An occupational therapist and speech-language pathologist mentioned that much of the learning came from medical residents presenting on palliative care research on the unit, and seminars on palliative care, respectively, to use as guides in approaching culture in practice. A group of participants discussed the need for an interprofessional network to navigate patients’ needs and gauge more insight into addressing cultural needs in practice. It is worth noting how one physiotherapist reflected on her experience working with different ethnic and racial backgrounds. Specifically, she alluded to providing care to the Indigenous community and learning more about their culture using spiritual care workers and chaplaincy as modes of assistance:

I would bring my team in here, because there was a lot of help – the chaplain we have; she really educated me on their culture. So, knowing about every culture is very difficult, right? Like, I come from a South Asian background, so I’m not very well-versed with many cultures here, but I have worked with them and that gives me an understanding of, you know, how they function, how they want things to be done, and what is- What- How they feel respected, you know? (RT-010, physiotherapist).

Finally, the organization of the unit, including having consistent allied health providers rather than rotating providers and being adequately staffed, helped build and maintain rapport with patients and understand their needs and preferences.

## Discussion

This study sought to identify how allied health providers understand and practice cultural humility. Eleven allied health providers were interviewed in this qualitative study. The following three themes, which speak to how allied health providers understand the concept of and practice cultural humility and how they navigate relationships with patients who are palliative or at EOL from cultures other than their own, identified from the data: (i) Interpreting and understanding of cultural humility in palliative and EOL care; (ii) Values, conflicts, and ethical uncertainty when practicing cultural humility at EOL; (iii) The ‘how to’ of cultural humility in palliative and EOL care. The findings of this study, including strategies used by providers to practice cultural humility, have the potential to improve patient experiences by supporting higher quality clinical interactions. In addition, the study findings may have implications on professional development and Canadian pre-qualifying/pre-licensure education related to health equity and cultural humility.

All providers in this study acknowledged the importance of cultural humility in palliative and EOL care. This is not surprising given widespread recognition that cultural humility is an aspect of effective palliative care [73], and it is a practice recommended by professional associations due to its associated benefits [52–54]. For instance, research suggests that providers who use culturally humble approaches can better understand and address patient needs, including the patient’s cultural beliefs, values, and practices of illness and death [33, 74, 75]. In addition, cultural humility may improve communication, trust between patients and providers, and care quality in palliative care [33]. Communication is vital to palliative care, but effective communication with providers is reported as the most prevalent unmet need [76, 77]. Improving communication through cultural humility may address unmet communication needs and potentially reduce culture-related healthcare disparities borne from providers’ lack of connection to patients [74].

An interesting facet of allied health providers’ experiences of practicing cultural humility was navigating professional and ethical challenges and conflicts. Providers shared multiple examples from clinical situations that they found challenging, and that impacted their ability to be culturally humble and made them feel uncertain about their approach. One of the most common challenges they reported was requests by family members for nondisclosure of the patient’s palliative status. As recognized in the literature, it is more common in some cultures for individuals to believe that revealing palliative status may harm the patient, and nondisclosure of diagnosis and prognosis and avoiding discussion about death and dying are common across some countries [26, 74, 78, 79]. These situations can lead to ethical and professional dilemmas and stress for health providers as they may not comply with their professional standards [79], particularly related to informed consent required for assessment and intervention. Challenges and conflicts encountered by allied health providers can require strategies and support beyond the intra-personal level. For example, relational strategies (e.g., collaboration with team [25]) and institutional policies/standards (e.g., adoption of communication tools to effectively communicate with patients and families while respecting the patient’s right to knowledge and preferences may be necessary) [26] can be required to support allied health providers to navigate such challenges and conflicts in practice.

Descriptions of cultural humility provided by allied health providers in this study aligned with common definitions [33, 57], despite many reporting that they did not receive education or training on cultural humility in their health profession’s educational curriculum. This finding reaffirms the need for cultural humility to be effectively integrated into pre-licensing education curriculums [25, 73]. In addition, it highlights the need for ongoing professional development education and effective knowledge translation approaches to adopt practice changes. Future research should address the lack of standardized training and education on cultural humility as doing so can support organizations and pre-qualifying/prelicensure curricula in providing relevant training and education [25, 73].

Health providers have been found to have the same levels of implicit bias as the general population [80]. Given this, it is imperative that these providers make conscious efforts to counter these biases. The results of this study indicate that the participant identified strategies for integrating cultural humility in practice described by Canadian allied health providers in our study align with previously identified strategies for cultural humility [73, 81]. For participants, cultural humility was not an additional or distinct practice; it appeared to be a practice and mindset interwoven within all aspects of their clinical activities. While many of the strategies could be adopted in any context, the clinical examples provided by participants in this study demonstrate how they have applied the strategies to palliative and EOL care contexts. For instance, providers highlighted the need for open-ended questions to explore death and dying preferences and engage in ongoing self-reflection to address their implicit bias [73]. Prior literature has suggested that bias, which involves implicit stereotyping and prejudices, reduces healthcare quality (e.g., poor patient-provider communication and patient satisfaction) [82, 83] and that health providers have the same levels of implicit bias as the general population [80]. Another recommended strategy in palliative care is self-awareness for health providers to explore their own perceptions about death and dying, which could help providers be open to learning about other perceptions that may differ from theirs [73].

Participants highlighted the need to consider intersectionality in cultural humility practices [84]. As culture is dynamic, rather than static and homogeneous, it is necessary to understand beliefs, practices, and values shaped by multiple factors and social identities, such as gender, history, geography, social norms, and power dynamics [85]. This understanding requires providers to re-think their conceptualization of culture, recognizing its complexity [84]. Finally, acting on cultural humility is enabled by practice systems and contexts, as organizational support is necessary for practice changes [86]. Allied healthcare providers can only engage in cultural humility practices if the infrastructure, resources, and systems exist to facilitate their efforts toward cultural humility.

### Strengths and limitations

This study was conducted remotely, which enabled cross-Canada recruitment and representation. In line with ID, investigators with personal and clinical experiences of palliative care considered how their interactions with allied health providers aligned or were contrary to the perspectives participants raised. However, there are notable limitations of this study. First, we were unable to recruit men or individuals of African ancestry in this study; their perspectives are not represented. Second, while we had a small sample size, we believe we had attained data sufficiency. Finally, while we strived to capture the perspectives of allied health providers, our sample comprised primarily occupational therapists. Further research may be necessary to capture the perspectives of other allied health providers not captured in this study.

## Conclusions

Allied health providers use multiple strategies, including intra-personal, inter-personal and contextual/health systems strategies, to manage relationships with patients from diverse cultural backgrounds and practice cultural humility. While ethical and professional conflicts and challenges can lead to uncertainty and stress, leveraging relational strategies and health system enablers can help allied health providers respond effectively to the conflicts and challenges. For example, ongoing professional development training may support allied health providers in practicing cultural humility to ensure patients’ needs and preferences are integrated into their palliative and EOL care.

## Electronic supplementary material

Below is the link to the electronic supplementary material.

Supplementary Material 1

## Data Availability

The datasets generated and/or analysed during the current study are not publicly available due privacy restrictions but may be available from the corresponding author on reasonable request.

## Abbreviations

ID: Interpretive Description
EOL: End-of-life
