# Supplementary material for: A qualitative exploration of allied health providers’ perspectives on cultural humility in palliative and end-of-life care
Source: BMC Palliat Care. 2023 Jul 12;22:92. doi: 10.1186/s12904-023-01214-4 (PMC10337104; doi:10.1186/s12904-023-01214-4)
Supplement: Supplementary file 1 — Supplementary Material 1 [file 12904_2023_1214_MOESM1_ESM.docx]

**Supplementary material - Interview guide**:

1. What came to mind when you thought of this topic, and what interested you in this study?
   1. Were there any specific examples that came to mind?
2. Can you please describe your role and the diversity of clients you work with?
   1. What does the word "culture" mean to you?

*Some have defined culture as broad and encompasses multiple intersecting factors like race, ethnicity, gender, age, differing abilities, sexual orientation, religion, spirituality, and socioeconomic status.*

1. How are a client and family’s cultural values, practices, and beliefs addressed by an allied health professional in EOL/palliative care?
   1. How do allied health professionals learn about and apply clients’ culture and cultural values, and beliefs at EoL?
      1. What questions would you ask a client?
      2. What discussions do you have with your team?
   2. Can you please provide an example of how client and family’s cultural values, practices and beliefs are factored in by allied health professionals in EOL/palliative care?
   3. What factors are important to consider for allied health professionals in EOL and palliative care working with clients of another race or ethnicity?
      1. Different genders, ages, differing abilities, sexual orientations, religions, spirituality, and socioeconomic status?
2. How would you describe culturally humble or sensitive care?
   1. What steps can be taken to create a sense of partnership with clients from another culture?
3. What do supportive interactions look like when working with clients of another culture?
   1. What organizational factors could help with working with clients from another culture (e.g., institutional, limited training)
   2. How do you navigate cultural differences with your clients?
4. Can you please tell me about a time when you had to navigate differences in culture or other identities when working with a client who was palliative and/or at the EoL?
5. What power differences could exist between allied health professionals and clients from another culture?
   1. How did you identify these differences and recognize the need to address them?
   2. Can you please share any decisions and actions you have used to recognize identify and address power imbalances?
   3. Can you describe any actions you took to overcome these power differences with a client in the past?
6. Can you describe an instance when you encountered a challenge in addressing the values or preferences of a client from another culture?
   1. How did you navigate this?
   2. How did you feel?
7. How can allied health professionals reduce negative outcomes (e.g. harmful, damaging, or destructive consequences such as discrimination, exclusion, inequity, disparities, prejudice, oppression, intolerance, stereotyping, stigma, and marginalization) in EOL and palliative care when working with clients from another culture?
8. What strategies would be helpful for new allied health professionals to effectively integrate a client and family’s viewpoint, belief, or lifeway in palliative/EOL RT?
   1. How do you emphasize respect and negotiate treatment plans?
9. What does self-reflection look like for you?
   1. Can you please provide some examples of what you mean by self-reflection?
10. How do you facilitate participatory decision-making with clients?
    1. What strategies do you employ to build strong relationships with culturally diverse clients?
11. Can you describe an instance where you feel like the clinician-client relationship could have been improved with a client from another culture? How did you improve the relationship?
    1. What advice would you give to a new allied health professional working in palliative care to deal with this type of instance?
12. What other barriers do/have you experienced in providing culturally humble care in palliative and EoL care settings?
13. What does the concept of cultural humility mean to you?

**Additional probes to prompt discussion during interview:**

- Can you tell me more about that?
- What was that like for you?
- Could you please clarify what that meant for you; could you walk me through that?
- What are some thoughts or feelings that you had?
